# Synthetic dataset of speckle images for fiber optic temperature sensor

**DOI:** 10.1016/j.dib.2023.109134

**Published:** 2023-04-07

**Authors:** Juan Arango, Victor Aristizabal, Francisco Vélez, Juan Carrasquilla, Jorge Gomez, Jairo Quijano, Jorge Herrera-Ramirez

**Affiliations:** a Instituto Tecnológico Metropolitano, Engineering Faculty, Medellín, Colombia; b Universidad Cooperativa de Colombia, Engineering Faculty, Medellín, Colombia. PhD Student EAFIT University; c Vector Institute, MaRS Centre, Toronto, ON M5G 1M1, Canada; d University of Waterloo, Department of Physics and Astronomy, ON N2L 3G1, Canada; e Politécnico Colombiano Jaime Isaza Cadavid, Basic Science Faculty, Medellín, Colombia

**Keywords:** Optical sensor, Modal interference, Specklegram, Fiber speckle

## Abstract

The published data correspond to images of simulated specklegrams, which result from the calculation of the modal interference that occurs in a multimode optical fiber. These have a characteristic pattern due to the constructive or destructive interference between the light modes depending on their phase differences. The specklegram contains valuable information since the propagation of the modes varies according to the influence of some external disturbances, and therefore, the speckle pattern changes. This dataset contains specklegrams that vary according to the temperature. These data have been obtained by simulation using the finite element method (FEM) through the COMSOL multiphysics platform. In the simulation, the vector wave equation is solved, and the refractive index of the fiber is recalculated due to the temperature change. We simulated a 1490 nm wavelength laser, an optical fiber with a core diameter of 50 µm and cladding diameter of 125 µm. The dataset contains specklegrams covering the range of temperatures from 0°C to 120°C in 0.2°C steps.

**Specifications Table**

**Table:** 

Subject	Optics
Specific subject area	Fiber optics sensors
Type of data	Images Python notebooks Matlab scripts
How the data were acquired	The data were acquired on a PC using Finite Element Method (FEM) simulation on the COMSOL multiphysics platform and Matlab.
Data format	Raw
Description of data collection	The specklegrams of this database were simulated by finite element method simulation (FEM), using discrete calculations for the propagation of several modes in the core and cladding of a multimode optical fiber. In this propagation, different light modes and their modal interference at the fiber output were calculated. This numerical solution is obtained with the vector wave equation and recalculating the refractive index that varies when the fiber undergoes a thermal change. This information was processed from MATLAB to adjust the COMSOL grid to a square grid and generate the speckle intensity matrix. These data were normalized with respect to the entire dataset, i.e., with respect to the minimum and maximum pixel value in all specklegrams.
Data source location	• Institution: Instituto Tecnológico Metropolitano • City/Town/Region: Medellín • Country: Colombia
Data accessibility	In this repository you will find the original dataset, the augmented dataset [1], the matlab code, the COMSOL file, and the python notebook codes for training and prediction with CNN. Repository name: OSF registries. Data identification number: 10.17605/OSF.IO/ZFMP5 Direct URL to data: https://osf.io/zfmp5/

## Value of the Data

• Free dataset of specklegrams corresponding to different temperature perturbations in an optical fiber.
• Researchers can test different analysis methods for fiber optic specklegram sensors, including some machine learning algorithms. With the later ones, it is possible to train a network that could later serve experimental specklegrams by fine tuning the training.
• Specklegram simulation facilitates the analysis of specklegrams for research purposes, due to the acquisition time in an experimental setup, the manual process to obtain each image or the automation of the process.
• The data are labeled with their respective parameters so that the experiment can be reproduced.
• These simulations and the replication of them can be used to study how modal interference affects the signal quality in a fiber. These simulations can help to identify the most critical factors that contribute to modal noise, such as the fiber geometry, the wavelength of light used, and the light launching conditions.

## 1 Objective

We designed this dataset to evaluate different analysis methods of speckle patterns (specklegrams) in fiber optic sensors and to provide a reference for researchers to test their speckle pattern generation models or experimental data. This dataset allows the assessment of analysis techniques and the models of the relationship between the physical variable (temperature) and the generated images. In this way, researchers from different fields as image processing, sensor design, or modeling of physical processes in fibers, can avoid the long and sometimes cumbersome process of experimental data acquisition.

## 2 Data Description

**Fig. 1:**
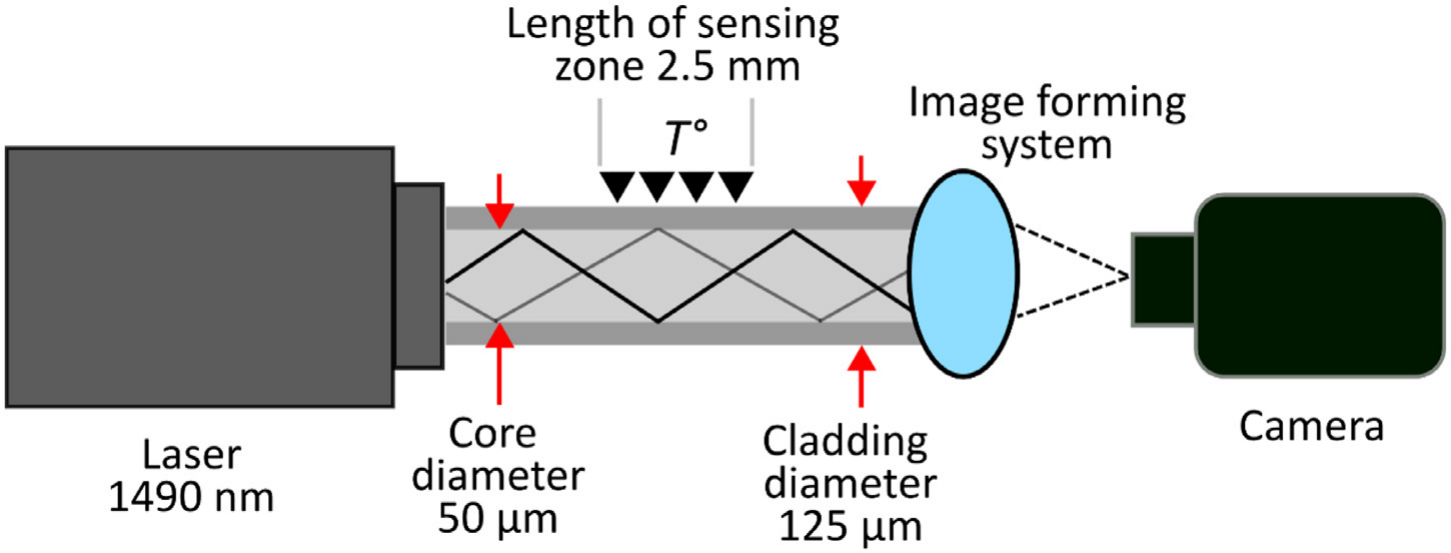
Representation of the assembly parameters used in simulation for the FSS.

**Fig. 2:**
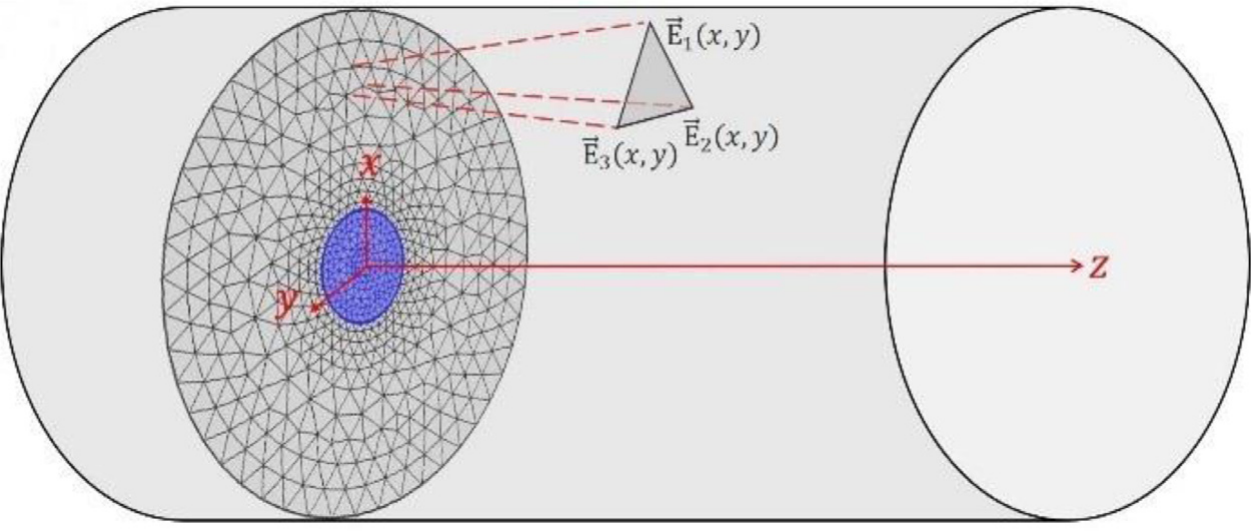
Discretization of the analysis domain defined for a MMF through a triangular mesh for the FEM implementation.

Modal interference is reflected in the speckle pattern that can be obtained at the output of a multimode fiber. This pattern contains important information, since the interference between the modes propagating through the fiber will be affected depending on some perturbations along the fiber [2], [3], [4]. There are different variables that can be measured by means of these specklegrams that affect the propagation of the modes, among them is the temperature. Systems that use these specklegrams to measure variables are called *Fiber Specklegram Sensors* (FSS) (see Fig. 1). The dataset of this paper contains different speckles that vary according to a thermal change in the fiber acquired by finite element simulation (FEM) (see Fig. 2).

Two datasets are presented: the original dataset and an augmented dataset version. The original dataset was simulated over a range of 0°C to 120°C with steps of 0.2°C, resulting in a total of 601 specklegrams. Each image has a dimension of 126 × 126 pixels.

The augmented dataset contains a total of 138230 specklegrams. This is composed of the 601 simulated images, from each of these 45 random rotations are performed, and from each original or rotated image 4 new ones are created with random Gaussian noise (this has a random variance value between 1 and 15).

**Fig. 3:**
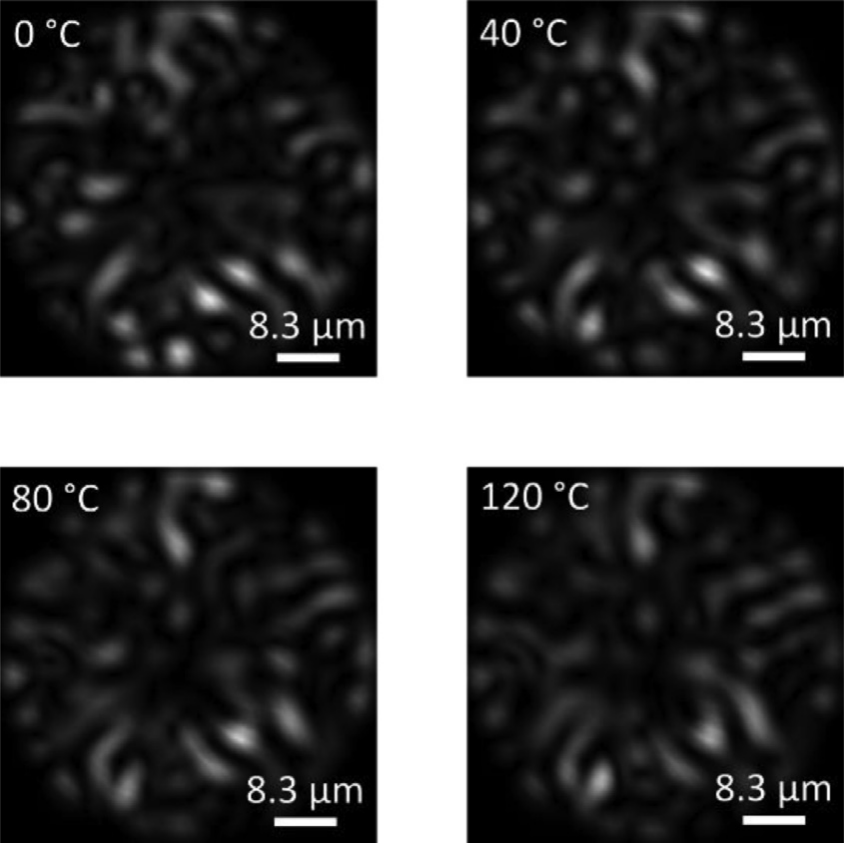
Examples of specklegrams from the original dataset.

Fig. 3 shows some examples of specklegrams acquired from the dataset at different perturbation temperatures.

**Fig. 4:**
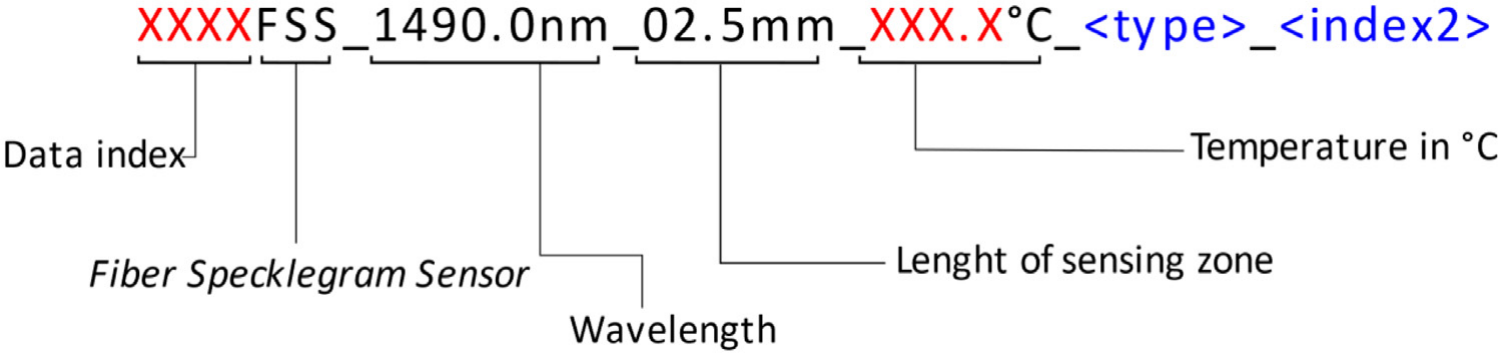
Nomenclature of the tiff images in the dataset. Note that the letter in red corresponds to the only elements that change in the file names (data index and temperature). Elements in blue only appear in the augmented dataset, *<type>* refers to one of three image types: S: simulated (original). SR: simulated and rotated. SRN: simulated, rotated and with Gaussian noise.

Fig. 4 explains the nomenclature of each of the images in the dataset, which are in grayscale with a depth of 8 bits and were saved in tiff format. In this dataset there are only specklegrams with the parameters, so the only thing that is modified in it are the temperatures.

Matlab script that calculates the refractive indices thermal effect and update them in the Comsol Multiphysics model contained in the Comsol file FG050LGA_MMF_fina.mph. Comsol is a multiphysics scientific software that models systems using the finite element method (FEM), which has a series of modules that help model specific physical phenomena (thermal, flow, electromagnetic, etc.). Here, a modal analysis was performed with the Wave Optics Module and using a MATLAB script (with the interfacing livelink for Matlab developed by COMSOL) to control and update the variation of the parameters and the sequence of the thermo-optical FEM executed in COMSOL.

## 3 Experimental Design, Materials and Methods

Synthetic specklegrams were obtained by computational simulation of the optical interference pattern, or Speckle, in a multimode optical fiber. The simulation computes the solution of the propagation of light for the electric field in an optical fiber as a waveguide. These speckle patterns are affected by changes in the refractive index of the optical fiber acting as a sensing device. Thus, as the optical fiber is affected by external disturbances, the speckle patterns exhibit changes useful for assessing the intensity of these disturbances.

**Fig. 5:**
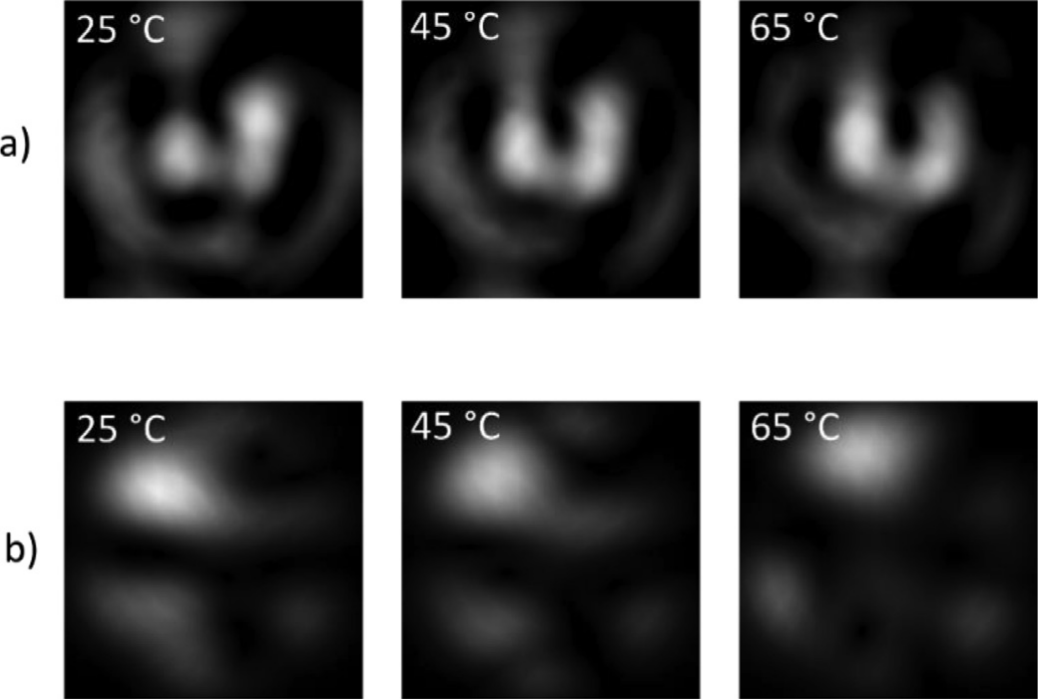
Example of experimental and simulated specklegrams for a fiber of 9 um core diameter, 0.14 numerical aperture, 12 mm sensing zone, and 633 nm laser wavelength. a) Experimental specklegrams, b) Simulated specklegrams.

Fig. 5 shows an example of experimental and simulated speckle patterns for a fiber optic of 9 µm core diameter, numerical aperture of 0.14, sensing length zone of 12 mm, and laser wavelength of 633 nm, submitted to three thermal disturbances. Fig. 5a presents the experimental specklegrams corresponding to temperatures of 25°C, 45°C, and 65°C, and Fig. 5b the simulations at those same temperatures. Note that the speckle distribution between the experimental and simulated specklegram varies, but the simulations show close behavior to the practical phenomenon regarding the evolution of the pattern with the change of temperature. The differences in the form of the speckles can be attributed to experimental conditions such as fiber bending or light launching conditions into the fiber. Further details of the specklegram simulation process can be found in previous work [4].

For the datasets presented in this work, the images correspond to synthetic speckle pattens of a step-index multimode optical fiber subjected to temperature changes over a range from 0°C to 120°C with 0.2°C steps. The calculations were developed using the Finite Element Method (FEM) in a numerical model implemented in the software COMSOL. There, a modal analysis was performed with the Wave Optics Module and using a MATLAB script (with the interfacing livelink for Matlab developed by COMSOL) to control the variation of the parameters and the sequence of the thermo-optical FEM executed in COMSOL (see more details of the models implemented in [5,4]). Table 1 shows the parameters carried out in the simulation of the dataset.

**Table 1:** Simulation parameters of the specklegram dataset.

Simulation parameters		
Mesh size	Min. 0.44 µm	Max. 1.88 µm
Material	Fused silica doped with P_2_O_5_ (phosphorus pentoxide)*	
Refractive index (without temperature disturbances)	Core	1.4447
	Cladding	1.4279
Thermo-optical coefficient	Core*	-10 × 10^−6^
	Cladding*	10.5 × 10^−6^
Numerical aperture	0.22	
Diameter	Core	50 µm
	Cladding	125 µm.
Wavelength		1490 nm
Length of the sensing zone		2.5 mm

⁎ Parameters taken from [6].

The length of the sensing zone refers to the length of the part of the fiber that is disturbed, in this case by thermal changes. If this length is greater, the changes in the speckle distribution will also be greater, meaning that the sensor would be more sensitive. This is shown in more detail in [4].

To obtain the speckle pattern in multimode optical fibers (MMF) under a given temperature, the electric field $\vec{E}$ of each mode that propagates in the FOM is calculated by solving the vector wave equation:

(1) $$\nabla \;\times \;\nabla \;\times \vec{E}-k_{0}^{2}n^{2}\vec{E}=0$$

where $k_{0}=2\pi /\lambda_{0}$ is the wave number in vacuum, $\lambda_{0}$ is the wavelength in vacuum and n is the refractive index tensor, which undergoes changes given by

(2) $$\Delta n_{i}=n_{i}-n_{0}=\Delta n_{i}^{TM}+\Delta n_{i}^{TO},\;\text{with}\;i=x,\;y,\;z$$

Here, $\Delta n_{i}^{TM}$ is the change in index due to thermo-mechanical disturbance (changes in the index due to material thermic expansion), and $\Delta n_{i}^{TO}$ the change caused by thermo-optical disturbance (direct index changes due to temperature variations). Then using (2) we obtain in approximation (see [3], [4]):

(3) $$n\approx n_{x}=n_{y}=n_{z}\approx n_{0}+C_{TO}\left(T-T_{0}\right)$$

where $C_{TO}$ is the thermo-optical coefficient, $T_{0}$ is the initial or reference temperature, and $n_{0}$ is the correspondent refractive index.

For a simplified calculation, if an infinite length optical fiber is assumed where the refractive index is invariant along the propagation axis, a 2D approximation of the problem is obtained. Here, the electric field $\vec{E}$ of each mode can be calculated by numerically solving Eq. (1) through the finite element method (FEM) (see more details in [5,[7], [8]). To calculate the field by FEM, the cross section of the fiber is discretized into triangular elements where Eq. (1) is solved in each of them and then they are assembled to obtain the general solution of each mode (See Fig. 2). In this way, the vector field of the modes and the propagation constants for the thermally disturbed system are obtained. All calculated modes are added vectorially to find the field of the speckle pattern [5].

## 4 Use of Dataset in a Deep Learning Interrogation Scheme

A dataset was analyzed through a deep learning architecture based on CNNs to extract features from a fiber specklegram sensor (FSS). The architecture implements the block structure of a VGG (Visual Geometry Group): conv-RELU → conv-RELU → MaxPooling, where each convolution layer has as output the RELU (Rectified Linear Unit) activation function. Subsequently, the regression process is performed to predict the temperature values associated with the speckle pattern of the simulated Speckle patterns.

The model was fitted using a dataset of 601 images corresponding to patterns associated with different temperatures ranging from 0°C to 120°C with 0.2°C steps. LE dataset was divided into 481 images for training and 120 for predictive testing and then 20% of the training data was taken for validation. Model training was performed in the Python programming language with the Keras and TensorFlow libraries. The learning rate was $8\;\times 10^{-5}$ with 300 epochs and a dropout of 50% was added to avoid overfitting.

The results obtained in the prediction with the dataset showed low RMSE errors, around 1.42°C, which for this type of sensing scheme represents an improvement in its metrological characteristics. (These codes are available in the same public repository of the dataset. See *Data accessibility* section)

## Ethics Statements

The authors declare that the present work did not include experiments on human subjects and/or animals.

## CRediT authorship contribution statement

**Juan Arango:** Software, Validation, Writing – original draft, Visualization. **Victor Aristizabal:** Methodology, Software, Data curation. **Francisco Vélez:** Methodology, Validation, Formal analysis. **Juan Carrasquilla:** Formal analysis. **Jorge Gomez:** Validation. **Jairo Quijano:** Writing – review & editing. **Jorge Herrera-Ramirez:** Writing – review & editing, Data curation, Methodology, Supervision.

## Declaration of Competing Interest

The authors declare that they have no known competing financial interests or personal relationships that could have appeared to influence the work reported in this paper.

## Data Availability

Synthetic dataset of fiber specklegram sensor with changes of temperature (Original data) (OSF).
